# How does it feel? Passage of time judgments in speeded RT performance

**DOI:** 10.1007/s00426-023-01854-4

**Published:** 2023-07-04

**Authors:** Daniel Bratzke, Arne Hansen

**Affiliations:** Department of Psychology, University of Bremen, Bremen, Germany

**Keywords:** Time perception, Passage of time judgments, Introspective RTs, Reaction time performance

## Abstract

The relationship between duration perception and the feeling of time passing (passage of time) is not yet understood. In the present study, we assessed introspective reaction times (RT) and passage of time judgments in a speeded RT task. Task difficulty was manipulated in a numerical comparison task by numerical distance (distance from the number 45) and notation (digit vs. word). The results showed that both effects were reflected in introspective RTs, replicating previous results. Moreover, passage of time judgments showed a very similar pattern, with slower passage of time for more difficult comparisons. These results suggest that in the millisecond range judgments of duration and passage of time largely mirror each other when participants introspect about their own RT performance.

In psychological research, the term “time perception” is often used as synonymous for the perception of duration (e.g., Grondin, 2010). However, time perception comprises a variety of temporal experiences such as simultaneity, temporal order, the subjective present, temporal continuity as well as duration perception (e.g., Pöppel, 1997). While all these phenomena have been investigated in humans intensively since the foundation of psychophysics by Fechner (1860; see also Wearden, 2016), another experience that is often implicitly subsumed within the others, namely the feeling about the passage of time has not been studied systematically until the beginning of this century (e.g., Ogden et al., 2011; Wearden, 2015).

Intuitively, one would probably assume that the perception of duration and the passage of time (as well as other phenomena like the subjective present) are tightly linked to each other (see e.g., Pöppel, 1997). The precise relationship, however, seems rather unclear. For example, if we say that “time passed slowly”, we usually refer to an event (e.g. a train ride) where the subjective duration appeared to be longer than the objective duration. Thus, there seems to be a “slower passage of time = longer duration”- relationship. By implication, this would mean that any manipulation that leads to a longer perceived duration (of the same event) should be associated with a slower passage of time. This inference, however, might be wrong when there is no indication that our perception differs from the objective duration (e.g., because no external clock is available).

A common theoretical assumption is that our perception of time is based on an “internal clock” with a pacemaker that elicits ticks and a counter mechanism that accumulates these ticks as a representation of time (e.g., Creelman, 1962; Gibbon et al., 1984; Treisman, 1963; Wearden, 2016; Zakay & Block, 1997). Many phenomena related to the subjective duration of events can be attributed to a variable pacemaker rate (e.g., due to differences in arousal; e.g., Matthews & Meck, 2016; Wearden, 2016). If we accept these theoretical assumptions, the relationship between pacemeaker rate and the feeling of how time passes again is rather unclear, as Wearden (2015) has put it:”When pacemaker speed is altered compared with normal, in which case does time ‘‘fly’’ and in which case does it ‘‘drag’’?” (p. 166).

In fact, over the last two decades research has provided evidence that duration and passage of time (POT) judgments are less related than one would assume (Droit-Volet & Wearden, 2016; Droit-Volet et al., 2017; Jording et al., 2022; Martinelli & Droit-Volet, 2022a; Ogden et al., 2011; Wearden, 2015; Wearden et al., 2014). For example, Droit-Volet and Wearden (2016) investigated the relationship between duration and POT judgments in students and elderly people in everyday life with an Experience Sampling Method. Participants were alerted several times during their normal day and performed a duration estimation task (stimulus durations: 350–1650 ms), a temporal production task (500, 1000, or 1500 ms), provided POT judgments, and responded to a number of affective and activity questions. The results showed no significant relation between duration and POT judgments. Droit-Volet et al. (2017) replicated these results for the seconds range, but could also show a relationship between the two types of judgments for longer durations in the range of minutes. Here, slower POT judgments were associated with shorter duration judgments.

In a recent study, Martinelli and Droit-Volet (2022b) investigated what factors can affect POT judgments in simple laboratory tasks, such as a short-term memory task (Exp. 1), viewing “emotional” pictures (Exp. 2), and viewing neutral pictures of different duration (black and white geometric patterns; Exp. 3). Their results showed that all factors under investigation (task difficulty, emotional valence, and stimulus duration) influenced POT judgments, irrespective of whether the to-be-judged time interval was in the seconds or minutes range. Specifically, less difficult tasks, more negative stimuli and longer stimuli were associated with a slower passage of time. The authors concluded that POT judgments can be affected by a number of factors, including the perception of duration. Which of these factors becomes effective, however, should depend on the saliency of the factors in the encountered context.

In the present study, we aimed to explore the relationship between duration perception and the feeling of the passage of time in the millisecond range. To this end, we replicated Experiment 1 from Corallo et al. (2008), who assessed estimates of speeded reaction times in a numerical comparison task. In this task, participants are presented with numbers in two different notations (arabic digits vs. number words; ranging from 21 to 69, excluding 45) and are asked to indicate whether the presented number is smaller or larger than 45. In Corallo et al., participants provided estimates of their reaction times (introspective RTs, IRTs) after each trial, and the results showed that IRTs reflected the effects of notation and numerical distance (close to/far from the reference number 45) on objective RTs. In the present study, we assessed POT judgments as well as IRTs in this paradigm and examined how these judgements are affected by notation and numerical distance and how they are related to RT.

Based on the results of Corallo et al. (2008) and a number of other IRT studies (e.g., Bratzke & Bryce, 2022; Bratzke & Janczyk, 2021), we expected that both difficulty effects (notation and numerical distance) on RT would be reflected in IRTs. But what is to be expected regarding POT judgments in this paradigm? One possibility is that an awareness of the passage of time does not even emerge in this time range (< 2–3 s; see Droit-Volet et al., 2017). Participants would then need to infer the passage of time from other factors. For example, it might be that participants always indicate a very fast passage of time because the task requires to respond as quickly as possible. IRTs and POT judgements should then show dissociative patterns. Alternatively, POT judgments could be influenced by task difficulty and/or stimulus duration as in Martinelli and Droit-Volet (2022b). According to their results, more difficult numerical comparisons (number words and close numerical distance) should lead to faster POT judgements, and longer stimuli, which is RT in the present study, should lead to slower POT judgments. Either these presumably opposing effects could cancel each other out, or one of them might dominate, for example due to differences in salience between stimulus duration and task difficulty.

## Method

### Participants

Sixty-two volunteers participated for course credit. Eleven participants were excluded from analyses because of high error rates in the numerical comparison task (> 25%). A power analysis indicated that 51 participants would be sufficient to detect a two-way repeated measures interaction with alpha = 0.05, power = 0.80 and a large effect size of η_p_^2^ = 0.14 (d_z_ = 0.40; see Langenberg et al., 2022). The mean age of the final sample was 25.4 years (*SD* = 4.9 years; 35 female, 15 male, 1 not specified). All participants provided informed consent prior to data collection in accordance with the 1964 Helsinki declaration and its later amendments.

### Apparatus and stimuli

The experiment was an online experiment and run on the participant’s individual computer. It was created in PsychoPy (Peirce et al., 2019) and hosted by Pavlovia (https://pavlovia.org). The following visual angles refer to a 13 inch display and a viewing distance of 60 cm. The number stimuli were arabic digits (1°) and number words (2.5°), ranging from 21 to 69 (except 45). A visual analogue scale (VAS, length: 12°) ranging from 0 to 1200 ms was used for IRT collection (with five ticks labelled “0 ms”, “300 ms”, “600 ms”, “900 ms”, and “1200 ms” from left to right). Another VAS of the same length and five ticks was used for POT judgments, labelled “very slowly” (1) at the left end and “very fast” (5) at the right end (as in Martinelli & Droit-Volet, 2022b).

### Tasks and procedure

In each trial, a number (digit or word) was presented in the center of the screen for 300 ms. Participants had to indicate as quickly and as correctly as possible whether the number was smaller (left mouse click) or larger (right mouse click) than the reference number 45. Immediately after the response, a VAS appeared on the screen and participants were asked to provide an estimate of their RT (“Please estimate how long it took you to respond.”) in the IRT condition, and a POT judgment in the POT condition (“How did time pass during this task?”). It was emphasized that both judgments referred to the interval between stimulus onset and the participant’s response. The intertrial interval was 500 ms. The experiment started with 10 practice trials (only numerical task), followed by 192 experimental trials (96 trials for each notation). The order of the temporal judgments was counterbalanced across participants. Half of them provided POT jugments in the first half of the experiment, whereas the others provided IRTs first.

### Data analysis

Numbers with a numerical distance of more than 12 (from the reference 45) were classified as “far”, all the others as “close”. For RT analyses, the data from both temporal judgment conditions were pooled. Trials with an erroneous response in the numerical comparison task were excluded (12.1%), as were trials with RTs deviating more than 2.5 *SD*s from the individual mean per numerical distance and notation (3.1% of correct trials). Separate ANOVAs were then conducted for RT and temporal judgments (IRT and POT) with the within-subjects factors numerical distance (close vs. far) and notation (digit vs. word). The ANOVA for the temporal judgments additionally included the within-subject factor type of judgment (IRT vs. POT). Additionally, for this analysis IRTs were transformed to the POT scale (i.e., an IRT of 200 ms would get the value 1, and an IRT of 1200 ms would get the value 5). To investigate the contribution of stimulus duration and task difficulty on temporal judgments, we ran a linear mixed effect (LME) model analysis with RT, numerical distance, notation, and type of judgment (IRT vs. POT) as predictors, and judgment as outcome variable. For this analysis all continuous variables (RT, IRT, and POT) were z-transformed. The fitted model included all main effects and interactions in the fixed effects structure and individual RT intercepts and slopes in the random effects structure. The R package LME4 (Bates et al., 2015) was used to fit LME models using REML, and *p*-values were derived using the Satterthwaite approximation (R package LmerTest; Kuznetsova et al., 2017).

## Results

**Fig. 1:**
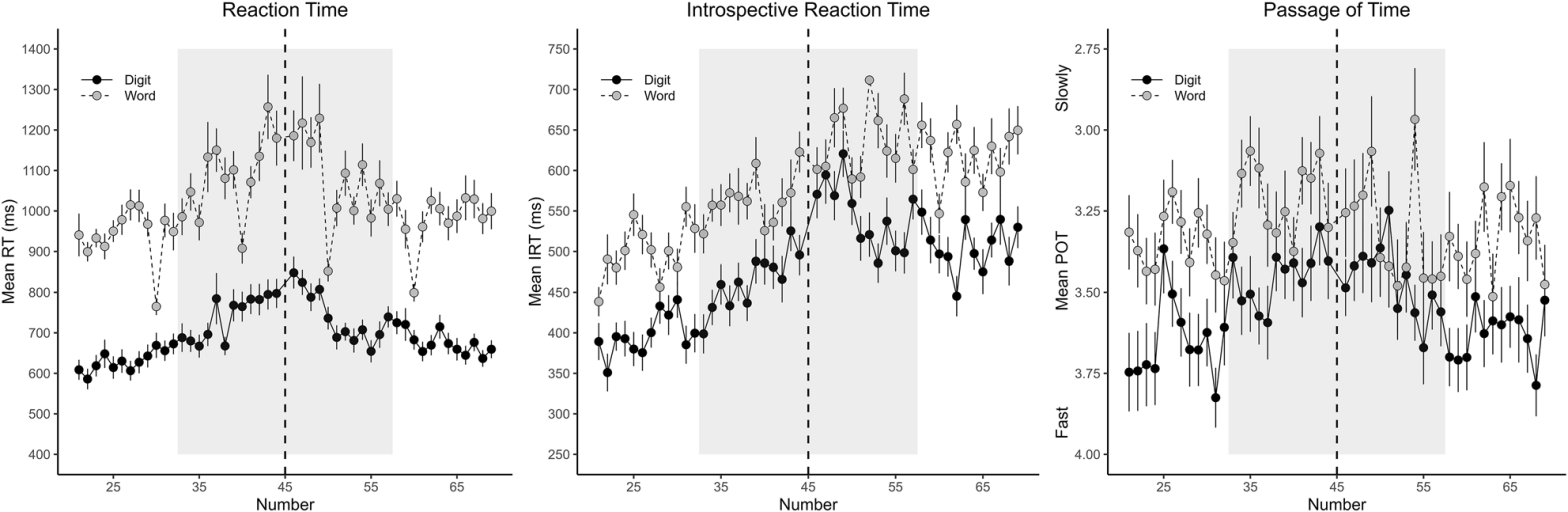
Reaction time (RT), introspective RT (IRT), and passage of time (POT) judgment as a function of number and notation. Numbers inside (outside) the shaded area were classified as close to (far from) the reference number 45 (dashed line). Note that for the sake of comparability (a) the IRT scale is stretched by the factor 2 compared to the RT scale, and the y-axis is reversed in the POT graph. Error bars represent ± 1 within-subjects *SE* according to Morey (2008)

The mean range of individual RT distributions was 1792 ms (*SD* = 1045 ms), with a mean minimum of 389 ms (*SD* = 97 ms) and a mean maximum of 2181 ms (*SD* = 1083 ms). The ANOVA for mean RT revealed significant main effects of numerical distance, *F*(1, 50) = 94.00, *p* < 0.001, η_p_^2^ = 0.65, and notation, *F*(1, 50) = 124.02, *p* < 0.001, η_p_^2^ = 0.71. RTs were longer for close numbers (895 ms) than for far numbers (801 ms), and also longer for words (1011 ms) than for digits (691 ms). The interaction did not reach significance, *F*(1, 50) = 3.98, *p* = 0.051, η_p_^2^ < 0.07. As can be seen in Fig. 1, IRTs and reversed POT judgments showed a similar pattern as RTs. For further analyses, the POT scores were therefore reversed (from 1 = very fast to 5 = very slowly). The ANOVA for the temporal judgments also showed significant main effects of numerical distance, *F*(1, 50) = 57.73, *p* < 0.001, η_p_^2^ = 0.54, and notation, *F*(1, 50) = 27.98, *p* < 0.001, η_p_^2^ = 0.37. The main effect of type of judgment was not significant, *F*(1, 50) = 3.09, *p* = 0.085, η_p_^2^ = 0.06. In contrast to the RT results, there was also a significant interaction between numerical distance and notation, *F*(1, 50) = 6.27, *p* = 0.016, η_p_^2^ = 0.11. For the temporal judgments, the effect of numerical distance was larger for digits than for words (0.018 vs. 0.011). Importantly, the three way-interaction between numerical distance, notation and type of judgment was not significant, *F*(1, 50) = 1.19, *p* = 0.281, η_p_^2^ = 0.02, just as the two-way interactions including type of judgment, both *p*s ≥ 0.257.

In addition to the numerical distance and notation effects, Fig. 1 suggests also an effect of numerical size, especially in the IRT pattern (middle panel). Therefore, we conducted additional individual regression analyses with numerical size as predictor and RT, IRT and POT as outcome variables. These analyses revealed significant positive standardized slopes for RT (*M* = 0.047, *p* = 0.004) and IRT (*M* = 0.255, *p* < 0.001) but not for POT judgments (*M* = 0.012, *p* = 0.739). This was confirmed by a one-way ANOVA comparing the slopes across all three outcome variables, which revealed a significant main effect of outcome variable, *F*(2, 100) = 19.03, *p* < 0.001, η_p_^2^ = 0.28. Post-hoc Tukey contrasts indicated significant differences between the RT and POT slopes (*p* < 0.001) and between the IRT and POT slopes (*p* < 0.001), but no difference between the RT and POT slopes (*p* = 0.684). It is important to note that these analyses relied on the combined RT data from both introspective (IRT and POT) conditions. The same regression analysis for RT based on only IRT and only POT trials showed significant slopes for IRT trials (*M* = 0.063, *p* = 0.002) but not for POT trials (*M* = 0.036, *p* = 0.053). A direct comparison of the slopes in the two instrospective conditions, however, did not yield a significant difference, *t*(50) = 1.44, *p* = 0.156.

**Fig. 2:**
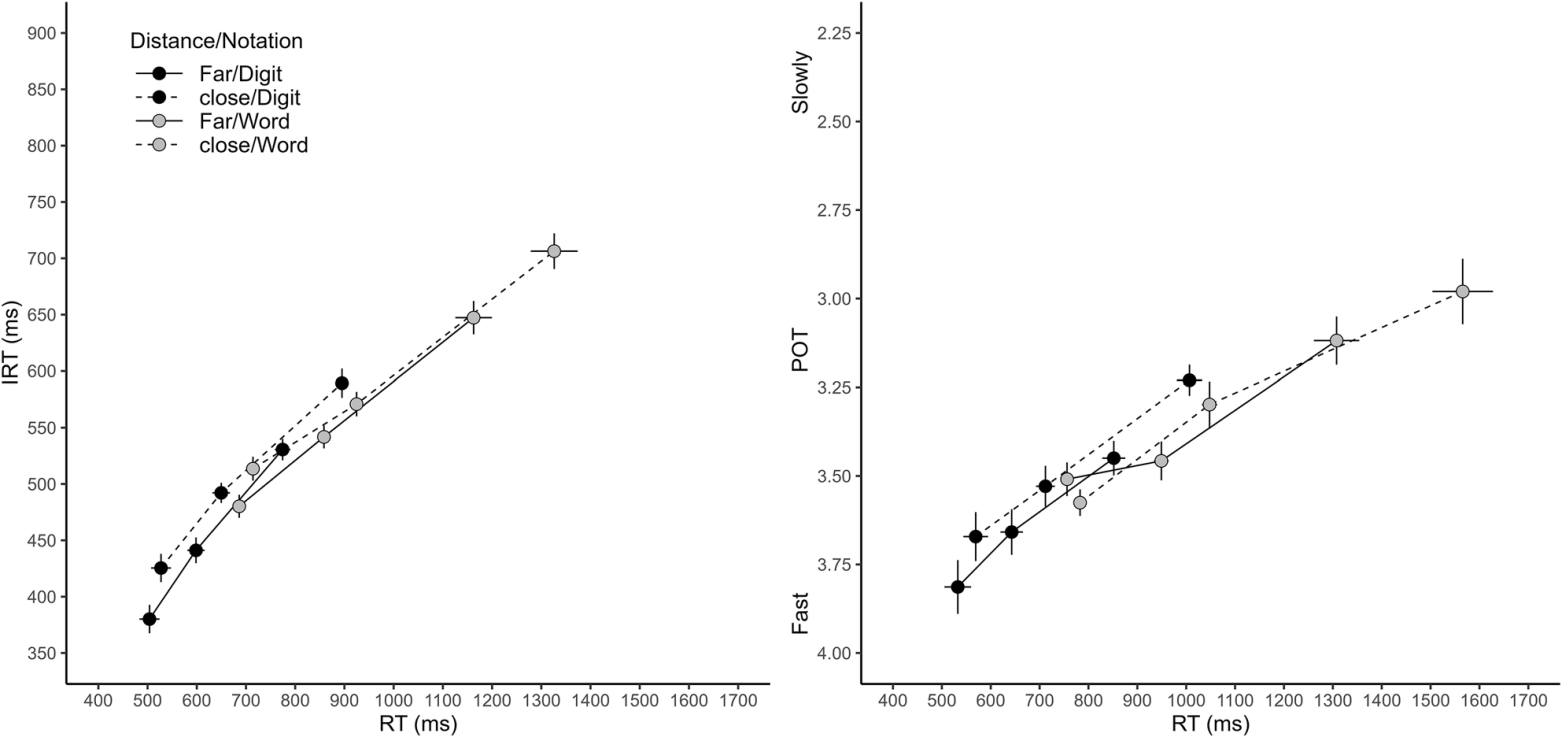
Relationship between reaction time (RT) and temporal judgments as a function of numerical distance and notation. Left panel: Introspective reaction time (IRT) plotted against RT (binned into three bins, vincentized). Right panel: Passage of time (POT) judgment plotted against binned RT. Error bars represent ± 1 within-subjects *SE* according to Morey (2008)

Figure 2 shows the relationship between RT and temporal judgments. The graphs of the functions for IRT and reversed POT judgments look strikingly similar. Accordingly, the LME model analysis yielded no significant main effect or interaction including the factor type of judgment, all *p*s ≥ 0.104. As expected, RT was a significant predictor, β = 0.58, *t*(48.7) = 11.61, *p* < 0.001. There were two other significant predictors, namely numerical distance, β = 0.07, *t*(8155) = 3.08, *p* = 0.002, and the interaction between RT and notation, β = 0.12, *t*(7960) = 4.18, *p* < 0.001, all other *p*s ≥ 0.297. As can be seen in Fig. 2, the graphs of the functions were shifted toward longer IRTs and slower POT jugments for close numbers. Addtionally, the slopes of the functions seem to be slightly steeper for digits than for words.

## Discussion

In the present study, we explored the experience of the passage of time in a speeded reaction time task. We were able to replicate the previous results by Corallo and colleagues (2008) that duration judgments (introspective RTs) reflected the effects of numerical distance and notation on RT. POT judgments showed a very similar pattern, with a slower passage of time for more difficult numerical comparisons (close vs. far numbers, words vs. digits). The LME model analysis revealed that both temporal judgments were influenced by numerical distance as well as objective task duration (i.e. RT), with relatively longer IRTs and slower POT judgments for more difficult comparisons.

Our results additionally showed an effect of numerical size on RT and IRT, with longer RTs and IRTs for comparisons with increasing size (i.e., the size effect; e.g. Verguts & Van Opstal, 2005), whereas there was no such effect for POT judgments. Importantly, although the size effect was much more pronounced in IRTs, it occurred also in RTs (a size effect can also be seen in both measures in Fig. 1 of Corallo et al., 2008). This suggests that the size effect in IRTs does not simply reflect a response bias induced by the numerical size of the presented numbers (e.g., a tendency to place the marker on the VAS more to the right for larger numbers corresponding with the increasing scale or tick labels of the VAS from left to right). Nevertheless, the size effect was significantly larger for IRT than for RT, and there was no significant difference between the effect for RT and POT judgments. This suggests that the numerical labeling of the VAS, or maybe even the numerical nature of the IRT estimate as such, might have played a role in the pronounced size effect in IRTs (there was no such labeling of the POT scale). Overall, it remains somewhat unclear to us how reliable the present size effects are, and how possible differences between IRT and POT results could be interpreted.

The result that difficult numerical comparisons were associated with a slower passage of time is in contrast to the results of Martinelli and Droit-Volet (2022b) in the seconds and minutes range with a different task. In their study, participants performed a series of working memory tasks (i.e., reproduction of 2- or 6-digit sequences) and were then asked to judge the passage of time. The series consisted of either 4 or 12 digit sequences and there was a fixed 500 ms response window, in which partcipants had to type in the sequence. This means that the interval to be judged included idle time as well as time filled with task performance, and the relationship between them was reciprocal. That is, the longer it took participants to respond in the working memory task the shorter the idle time in between subsequent tasks. Unfortunately, the authors only reported performance accuracy; however, it is very likely that more difficult and longer sequences resulted in longer RTs as well as typing times. As a consequence, the POT judgments in their study might be more related to the differences in idle time than to the difficulty of the task. A second possibility why we observed a different effect of task difficulty on POT judgments is that our difficulty mainpulations also affected the to be judged time interval, whereas it was constant in Martinelli and Droit-Volet’s study. As a third possibility, the longer overall task processing time in their study compared to the millisecond RTs in our study might have induced a qualitatively different experience of the passage of time. As already mentioned in the Introduction, it is possible that an awareness of the passage of time does not emerge in the millisecond range and therefore participants need to infer it from other factors, as for example, from IRTs.

In contrast to the effects of task difficulty, the present results seem to be consistent with Martinelli and Droit-Volet’s (2022b) observations regarding stimulus duration. In their study, longer stimulus duration and a longer time range (minutes vs. seconds range) resulted in slower POT judgments. In the present study, we considered RT rather than the presentation duration of the number stimuli, which was always 300 ms, as stimulus duration. Nevertheless, POT judgments were clearly affected by RT in the same direction as in Martinelli and Droit-Volet’s study. Since in the present study task difficulty also affected the “stimulus duration”, one might suspect that stimulus duration was the only factor affecting POT judgments. The LME model analysis, however, showed that POT judgments were affected by numerical distance beyond the POT-RT relationship, and the effect was still opposite to the difficulty effect in Martinelli and Droit-Volet’s study.

Previous studies have suggested that duration estimates and POT judgments can be distinguished, when time intervals in the seconds range are to be judged (e.g., Droit-Volet & Wearden, 2016; Droit-Volet et al., 2017), but that they are related when longer intervals in the minutes range are considered (Droit-Volet et al., 2017; Martinelli & Droit-Volet, 2022a). The present results clearly show that duration and POT judgments are very similar, if not interchangeable, when the to be judged temporal interval is very short (in the millisecond range) and filled with active task processing. This suggests that both temporal judgments rely on the same underlying mechanism in this time range. Possibly, in this situation POT judgments are inferred from duration judgments. However, it is also possible that both judgments are inferred or at least biased from factors other than temporal information, as for example from the feeling of difficulty or the knowledge about task difficulty (see e.g., Bratzke & Bryce, 2019; Bryce & Bratzke, 2014).

The present research is subject to several limitations. First, the durations we studied were filled with task processing and task difficulty directly affected the to be judged durations. This situation differs from more common time perception paradigms, in which participants judge the duration of a fixed set of durations (conveyed by a certain stimulus), which they perceive more or less passively. It remains an open question whether the present results generalize to this rather passive perceptual timing task. Second, we were not able to directly analyse the relationship between IRTs and POT judgments, for example, via correlational analyses. This was not possible because we did not assess the two measures within the same trial to avoid possible response biases (e.g. a tendency to indicate the same position on the two scales). Third, one could argue that the VAS method used in the present study is not one of the standard methods to assess duration estimates (as e.g. verbal estimation or temporal reproduction; see Grondin, 2008). However, we consider the VAS as a variant of the verbal estimation method (the estimate is indicated on the scale instead of typed in or spoken aloud), and a previous introspective RT study by Bryce and Bratzke (2015) yielded very similar results with the VAS and the temporal reproduction method. We are therefore reasonably confident that the present duration estimates are not dependent on the use of the VAS method. Finally, in the present study we used a prospective timing task, that is, participants knew in advance that they had to provide temporal judgments after each numerical comparison. Results might be different in a retrospective paradigm, where participants do not know about the temporal task in advance and therefore likely do not attend to time to the same degree. Studying the retrospective paradigm is of course rather uneconomic, especially with rather short durations. Nevertheless, Martinelli and Droit-Volet (2022a) investigated duration and POT judgments with relatively short (20–45 s) and long (80–180 s) durations (viewing an image) under prospective and retrospective conditions. They observed that the more participants attended to time the more slowly time seemed to pass and that even in the retrospective condition participants indicated that they had paid attention to time, even though to a lesser extend (a difference of about 0.3 on a 7-point Likert scale between the pro- and retrospective condition for short durations).

In conclusion, the present study yielded very similar results for duration estimates and POT judgments when participants judged their own reaction time performance in a numerical comparison task. The effects of task difficulty were reflected in both temporal judgments, with longer duration estimates and slower POT judgments for more difficult numerical comparisons. The present results suggest that these two different aspects of temporal experience (duration vs. passage of time) are almost indistinguishable in the millisecond range, at least when participants judge their own reaction time in a cognitive task.

## Data Availability

The datasets generated during and/or analysed during the current study are available from the corresponding author on reasonable request. This study was not preregistered.
